# Supplementary material for: Robust Relative Error Estimation
Source: Entropy (Basel). 2018 Aug 24;20(9):632. doi: 10.3390/e20090632 (PMC7513150; doi:10.3390/e20090632)
Supplement: Supplementary file 1 [file entropy-20-00632-s001.zip › supplement/rree-manual.pdf]

Package 'rree'

August 14, 2018

Type Package

Title Robust Relative Error Estimation

Version 0.2

Date 2018-08-10

Author Kei Hirose

Maintainer Kei Hirose <mail@keihirose.com>

Imports Rcpp

LinkingTo Rcpp, RcppArmadillo

Depends MASS, GeneralizedHyperbolic

Description An R package ``rree'' robust estimation via negative gamma-likelihood.
The MM algorithm is used.

License GPL (>= 2)

URL http://www.keihirose.com

NeedsCompilation yes

R topics documented:

rree 1
rree.generator 3

Index 5

`rree` *Robust Relative Error Estimation.*

Description

This function gives us the robust least product relative error (LPRE) estimator and robust least squares relative error (LSRE) estimator via gamma-likelihood from a "rree" object for fixed value of gamma.

Usage

```
rree(x, y, intercept=TRUE, gam, beta.init, type="LPRE", algorithm="MM",
     maxit=1000, cov.beta = TRUE, tol=sqrt(.Machine$double.eps))
```

Arguments

x	Matrix of predictors.
y	Vector of responses.
intercept	If TRUE, intercept is included.
gam	The value of gamma used in the gamma-likelihood function. If gam=0, the ordinary relative error loss function is minimized (i.e., likelihood function is maximized).
beta.init	Initial value fo beta. If missing, it becomes zero vector.
type	Either "LPRE" or "LSRE". Details are presented in our paper.
algorithm	"MM" "BFGS", or "hybrid". With the hybrid, first MM algorithm is conducted, and then BFGS is used. The hybrid is generally fast and stable. The BFGS is the fastest but sometimes unstable. The convergence of the MM is slow but MM is generally stable.
maxit	Maximum number of iterations in the MM algorithm.
cov.beta	If TRUE and type="LPRE", the asymptotic covariance matrix of estimator is computed.
tol	Threshold used for convergence of parameter.

Value

beta	Coefficient vector.
loss	The value of loss criterion.
cov.beta	Asymptotic covariance matrix of estimator.
weight	Weights in the weighted log-likelihood.
intercept	If TRUE, intercept is included.
x	Matrix of predictors.
y	Vector of responses.

Author(s)

Kei Hirose
<mail@keihirose.com>

References

Hirose, K. and Masuda, H. (2018). Robust relative error estimation. *manuscript*.

See Also

`predict.rree` and `rree.generator` objects.

Examples

```
#generate data
dat <- rree.generator(n=50, p=5, outlier.ratio=0.1) #about last 10% data values are outliers

#fitting
fit <- rree(dat$x, dat$y, gam=0) #ordinary LPRE
fit2 <- rree(dat$x, dat$y, gam=0.2) #robust LPRE with gamma=0.2
```

```
fit
fit2

#prediction
predict(fit2, newx=dat$x[1:10,])
```

---

rree.generator *Generate Dataset.*

Description

This function generates dataset with outliers. The target distribution is positive harmonic distribution or log normal distribution, and the contaminated distribution is log normal distribution.

Usage

```
rree.generator (n = 100, p = 20, type="rgig", seed=0, beta.true, x.sigma, x,
                sd.lnorm=1, outlier.ratio=0.1, outlier.par=c(2.5,0.1))
```

Arguments

n	The number of observations.
p	The number of variables.
type	Either "rgig" or "lnorm". "rgig" corresponds to the positive harmonic distribution, which is a density function for LPRE loss function. "lnorm" is the log-normal distribution.
seed	Seed for generating the random number.
beta.true	True value fo beta. If missing, `beta.true = rep(1/(p + 1), p + 1)`.
x.sigma	Covariance matrix of design matrix. If missing, the covariance structure is AR(1) with AR parameter 0.7.
x	Design matrix. If missing, `x = mvrnorm(n, mu = rep(0, p), Sigma = x.sigma)`.
sd.lnorm	Only used when target = "lnorm". Standard deviation of log normal distribution for target distribution (`sdlog` in `rlnorm`).
outlier.ratio	Outlier ratio.
outlier.par	Parameter vector for log-normal distribution for contamination of `meanlog` and `sdlog` in `rlnorm`.

Value

x	Matrix of predictors.
y	Vector of responses.
beta	coefficient vector.
x.sigma	Covariance matrix of design matrix.
n.true	The number of observations drawn from target distribution.
n.cont	The number of observations drawn from contaminated distribution.

Author(s)

Kei Hirose
<mail@keihirose.com>

References

Hirose, K. and Masuda, H. (2018). Robust relative error estimation. *manuscript*.

See Also

`rree` object.

Examples

```
#generate data
dat <- rree.generator(n=50, p=5, outlier.ratio=0.1) #about last 10% data values are outliers
```

Index

predict.rree *(rree)*, 1

rree, 1
rree.generator, 3
