# Supplementary material for: Robust Relative Error Estimation
Source: Entropy (Basel). 2018 Aug 24;20(9):632. doi: 10.3390/e20090632 (PMC7513150; doi:10.3390/e20090632)
Supplement: Supplementary file 1 [file entropy-20-00632-s001.zip › supplement/sample program.html]

Sample program of rree package


# Sample program of rree package

# Summary

The package `rree` implements robust relative error estimation. The function `rree.generator` generates data from positive harmonic distribution, which corresponds to the loss function of the LPRE. We can also generate outliers with arbitrary outlier ratio by changing the argument `outlier.ratio`. To fit the model, we use the `rree` function. The function `predict` is used to make a prediction. For detail, please refer to the help of `rree` function.

```
library(rree)
```

```
## Loading required package: MASS
```

```
## Loading required package: GeneralizedHyperbolic
```

```
#generate data
dat <- rree.generator(n=50, p=5, outlier.ratio=0.1) #about last 10% data values are outliers

#fitting
fit <- rree(dat$x, dat$y, gam=0) #ordinary LPRE
fit2 <- rree(dat$x, dat$y, gam=0.2) #robust LPRE with gamma=0.2
fit
```

```
## 
## Call: rree(x = dat$x, y = dat$y, gam = 0) 
## 
## beta:
## [1]  0.36766  0.25202  0.39204 -0.03252 -0.09134  0.52541
## 
## weight:
##  [1] 0.02 0.02 0.02 0.02 0.02 0.02 0.02 0.02 0.02 0.02 0.02 0.02 0.02 0.02
## [15] 0.02 0.02 0.02 0.02 0.02 0.02 0.02 0.02 0.02 0.02 0.02 0.02 0.02 0.02
## [29] 0.02 0.02 0.02 0.02 0.02 0.02 0.02 0.02 0.02 0.02 0.02 0.02 0.02 0.02
## [43] 0.02 0.02 0.02 0.02 0.02 0.02 0.02 0.02
```

```
fit2
```

```
## 
## Call: rree(x = dat$x, y = dat$y, gam = 0.2) 
## 
## beta:
## [1]  0.19799  0.38429  0.35980  0.18089 -0.06353  0.25913
## 
## weight:
##  [1] 0.0200894 0.0247332 0.0198692 0.0193874 0.0248939 0.0249067 0.0193572
##  [8] 0.0203329 0.0179628 0.0267462 0.0246585 0.0223991 0.0214285 0.0192457
## [15] 0.0203681 0.0291554 0.0251925 0.0190952 0.0210561 0.0180471 0.0165067
## [22] 0.0173746 0.0246328 0.0272392 0.0199662 0.0140033 0.0210588 0.0344358
## [29] 0.0208942 0.0239129 0.0232463 0.0225034 0.0175451 0.0118743 0.0165469
## [36] 0.0219116 0.0203814 0.0216468 0.0234695 0.0215921 0.0213107 0.0180821
## [43] 0.0221992 0.0209106 0.0226583 0.0237275 0.0034467 0.0054429 0.0019575
## [50] 0.0005974
```

```
#prediction
predict(fit2, newx=dat$x[1:10,])
```

```
##  [1] 0.8400173 0.3522975 1.9229040 3.2875169 0.7920715 0.9300295 2.6844779
##  [8] 1.7990998 2.6894584 0.5445647
```
